# Compared with conventional PCR assay, qPCR assay greatly improves the detection efficiency of predation

**DOI:** 10.1002/ece3.6494

**Published:** 2020-06-27

**Authors:** Ting‐bang Yang, Jie Liu, Jian Chen

**Affiliations:** ^1^ Key Laboratory of Southwest China Wildlife Resources Conservation (Ministry of Education) Institute of Ecology China West Normal University Nanchong China; ^2^ The State Key Laboratory of Biocatalysis and Enzyme Engineering of China School of Life Sciences Hubei University Wuhan China; ^3^ School of Nuclear Technology and Chemistry & Biology Hubei University of Science and Technology Xianning China

**Keywords:** conventional PCR, molecular gut content analysis, predation, qPCR

## Abstract

Studies of predation can contribute greatly to understanding predator–prey relationships and can also provide integral knowledge concerning food webs and multi‐trophic level interactions. Both conventional polymerase chain reaction (cPCR) and quantitative PCR (qPCR) have been employed to detect predation in the field because of their sensitivity and reproducibility. However, to date, few studies have been used to comprehensively demonstrate which method is more sensitive and reproducible in studies of predation. We used a *Drosophila melanogaster‐*specific DNA fragment (99 bp) to construct a tenfold gradient dilution of standards. Additionally, we obtained DNA samples from *Pardosa pseudoannulata* individuals that fed on *D. melanogaster* at various time since feeding. Finally, we compared the sensitivity and reproducibility between cPCR and qPCR assays for detecting DNA samples from feeding trials and standards. The results showed that the cPCR and qPCR assays could detect as few as 1.62 × 10^3^ and 1.62 × 10^1^ copies of the target DNA fragment, respectively. The cPCR assay could detect as few as 48 hr post‐feeding of the target DNA fragment. But the qPCR assay showed that all spiders were positive after consuming prey at various time intervals (0, 24, 48, 72, and 96 hr). A smaller proportion of the technical replicates were positive using cPCR, and some bands on the agarose gel were absent or gray, while some were white and bright for the same DNA samples after amplification by cPCR. By contrast, a larger proportion of the technical replicates were positive using qPCR and the coefficients of variation of the *Ct* value for the three technical replicates of each DNA sample were less than 5%. These data showed that qPCR was more sensitive and highly reproducible in detecting such degraded DNA from predator's gut. The present study provides an example of the use of cPCR and qPCR to detect the target DNA fragment of prey remains in predator's gut.

## Graphical abstract

Studies of predation can contribute greatly to understanding predator–prey relationships and can also provide integral knowledge concerning food webs and multi‐trophic level interactions. It is particularly important to choose applicable methods to identify predator–prey interactions. This manuscript offers a comparison of cPCR and qPCR assays in molecular gut content analysis.

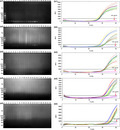

## 1 INTRODUCTION

Predation is a process in which an organism consumes all or part of the body of another living organism and directly obtains nutrients to maintain its nutritional homeostasis (Ge, 2008). Studies of predation can contribute greatly to understanding predator–prey relationships and can also provide integral knowledge regarding food webs and multi‐trophic levels interactions, which in turn influence ecological processes such as niche partitioning and interspecific competition (Burgar et al., 2014; Steele, Yi, & Zhang, 2018; Ylönen, Haapakoski, Sievert, & Sundell, 2019). In addition, studies of predation can screen for the main predators of target insect pest species as potential biological agents (Yang, Liu, Yuan, Zhang, Li, et al., 2017; Yang, Liu, Yuan, Zhang, Peng, et al., 2017). Therefore, it is important to develop an accurate technique to detect the interactions between predator and prey in the ecological and agricultural fields.

Molecular gut content analysis is the most common method for identifying predator–prey relationships in the field because of its sensitivity, specificity, and reproducibility (King, Read, Traugott, & Symondson, 2008; Macías‐Hernández et al., 2018). Conventional polymerase chain reaction (cPCR) assays have successfully qualitatively evaluated the predation of target prey (usually insect pests) by predators (Agustí, De Vicente, & Gabarra, 2000; Cuthbertson, Fleming, & Murchie, 2003; Harwood et al., 2007; Symondson, 2002). However, cPCR requires further treatment for visualization, which is time consuming and sometimes leads to carryover DNA contamination (Aslanzadeh, 2004). In addition, cPCR cannot quantify the amount of prey DNA using PCR products amplified from the predator's gut. To address these drawbacks, quantitative PCR (qPCR) assays were used to detect predation in the field (Matejusova et al., 2008; Troedsson, Simonelli, Nägele, Nejstgaard, & Frischer, 2009; Wang, Wang, Qiao, Zhu, & Cheng, 2013; Zhang, Lü, Wan, & Lövei, 2007). This method does not require post‐PCR manipulations, which is greatly time efficient and reduces the possibility of carryover contamination. In addition, the amount of prey DNA amplified from the predator's gut can be quantified by qPCR. However, in recent years, both cPCR and qPCR have been employed to detect predation in the field based on prey‐specific primers (Albertini et al., 2018; Cuende et al., 2017; Furlong, Rowley, Murtiningsih, & Greenstone, 2014; Li et al., 2017; Yang, Liu, Yuan, Zhang, Li, et al., 2017; Yang, Liu, Yuan, Zhang, Peng, et al., 2017). Although qPCR has been shown to be more sensitive and reproducible than cPCR in disease diagnosis (Paiva‐Cavalcanti, Regis‐da‐Silva, & Gomes, 2010; Sonawane & Tripathi, 2013), few studies have specifically demonstrated which method is more sensitive and reproducible in studies of predation; as far as we are concerned, only one study has shown that qPCR improved sensitivity compared with cPCR in molecular gut content analysis (Gomez‐Polo et al., 2015). Thus, more studies are apparently needed to compare the sensitivity and reproducibility of cPCR and qPCR assays in studies of predation.

*Pardosa pseudoannulata* (Araneae, Lycosidae) is a common predator of insect pests in the agroecosystem (Maloney, Drummond, & Alford, 2003). *Drosophila melanogaster* (Diptera, Drosophilidae) has often been used as food for spiders in the lab (Jing, Zhou, Du, & You, 2012). Therefore, they are readily available as materials to explore the sensitivity and reproducibility of cPCR and qPCR assays in studies of predation. We obtained DNA samples from *P. pseudoannulata* individuals that fed on *D. melanogaster* with the use of tenfold gradient dilution of standards (obtained from purified plasmid DNA). Both cPCR and qPCR assays were used to detect DNA samples from spider feeding trials and tenfold gradient dilution of standards. The result of this study provides an important reference for choosing applicable methods to identify the interactions between predators and prey in the ecosystem.

## 2 MATERIALS AND METHODS

### 2.1 Feeding trials

To compare the sensitivity of cPCR with qPCR in the detection of predation, separate feeding trials were carried out using adult female *P. pseudoannulata* and adult *D. melanogaster*. *Pardosa pseudoannulata* was collected at the wetlands along the Xihe River in Nanchong city, China. Individual spiders were reared in the lab using glass tubes with external diameter of 20 mm and length of 100 mm (Yongming experimental equipment factory, China), and only provided moistened sponges in the bottom of glass tube to ensure humidity. *Drosophila melanogaster* was reared in glass tubes with external diameter of 40 mm and length of 100 mm using the culture medium. The component of culture medium was referred to Bian, Yuan, Wang, and Qu, (2012). All spiders used in the experiment were starved at least a week in the lab (greenhouse conditions: 25 ± 1°C, 80%−85% relative humidity, L12:D12 hr photoperiod) prior to the start of the experiment. After starving, individual spiders were allowed to feed on three adult *D. melanogaster* within 1 hr in glass tubes with external diameter of 20 mm and length of 100 mm. Individual spiders that were observed to feed on all three fruit flies within 1 hr were used in the experiment. After feeding, the spiders at post‐feeding intervals of 0, 24, 48, 72 and 96 hr were used to test the sensitivity of cPCR and qPCR. Five individual spiders were used for each post‐feeding interval. Finally, spiders were placed individually in micro‐centrifuge tubes (1.5 ml) with 100% ethanol, stored at –80°C, and later used for DNA extraction.

### 2.2 DNA extraction

The genomic DNA of spiders from each feeding interval was extracted individually using a DNeasy Blood & Tissue Kit (Qiagen). We used whole spider specimen to extract genomic DNA. To avoid contamination, the extraction desk and instrument were scrubbed with 75% ethanol, and the spider was cleaned with ultra‐pure water before extraction. Extraction process referred to the manufacturer's instructions; ultra‐pure water was used to substitute for the spider as a negative control for each extraction process. The DNA of each extraction was eluted in 150 μl of the manufacturer's elution buffer. After extraction, the DNA samples were stored at –80°C and later used for detection.

### 2.3 Design of primers and TaqMan minor groove binder (MGB) probe

We followed the rules on primer design outlined by King et al., (2008). Shorter amplicons < 300 bp should be targeted wherever possible as the DNA molecules are broken into smaller fragments during digestion in the predator's guts. The primer pair COI‐F (5′‐CGATCAACAGGAATTTCATTAG‐3′) and COI‐R (5′‐TCCTGCTAGTACTGGAAGTG‐3′) was designed using a fragment of the cytochrome oxidase I (COI) gene of *D. melanogaster* from GenBank (554 bp, GenBank accession no. EF153615.1). Primers were designed using the Primer Express 2.0 software (Applied Biosystems).

The TaqMan MGB probe (5′‐CCTTTATTTGTTTGATCAGTAG‐3′) for *D. melanogaster* target DNA fragment was designed using the Primer Express 2.0 software. The probe was synthesized by Shanghai Bioligo Biotechnology Ltd.

### 2.4 The annealing temperature and specificity of primers

The annealing temperature of the primers was critical for determining PCR amplification efficiency (King et al., 2008). A temperature gradient PCR was used to determine the optimum annealing temperature of the primers. The genomic DNA of *D. melanogaster* was amplified by a CFX Connect™ Real‐Time PCR Detection System (Bio‐Rad, USA) using the designed primers described above. The amplification was carried out in a final volume of 20 μl. Each tube contained 10 μl *TransStart* ^®^ Probe qPCR SuperMix (Beijing TransGen Biotechnology Co. Ltd, China), 1 μl sample DNA, 1 μl reverse primer (10 μM), 1 μl forward primer (10 μM), 0.4 μl fluorogenic probe (10 μM), and 6.6 μl ultra‐pure water. The thermal cycle consisted of an initial step of 30 s at 94°C, followed by 40 cycles of 5 s at 94°C and 30 s at temperature gradient of 63.0°C, 62.5°C, 61.5°C, 59.7°C, 57.6°C, 55.8°C, 54.6°C, and 54.0°C. Data acquisition and analysis were carried out using the Bio‐Rad CFX Manager 3.1 software (Bio‐Rad).

To confirm that the designed primers (COI‐F and COI‐R) did not amplify the non‐target genomic DNA of *P. pseudoannulata*, we tested the specificity of the primers using genomic DNA from *D. melanogaster* and *P. pseudoannulata* (starved at least a week). DNA samples were amplified by a CFX Connect™ Real‐Time PCR Detection System using the designed primers described above. PCR conditions were as described above. The thermal cycle consisted of an initial step of 30 s at 94°C, followed by 40 cycles of 5 s at 94°C and 30 s at 57.6°C. Each run contained a non‐template control (without any nucleic acid). Each sample was assayed in triplicate. Data acquisition and analysis were as described above.

### 2.5 Standards

The target DNA fragment was cloned into the PUC57 vector (Shanghai Bioligo Biotechnology Co. Ltd). The recombinant plasmid DNA was propagated in DH5α competent cells (Beijing TransGen Biotechnology Co. Ltd). The bacteria were cultured in solid medium (LB/Amp [100 μg/ml]), and white colonies were obtained. The white colonies were inoculated into liquid medium (LB/Amp [100 μg/ml]). Finally, the DNA was purified using an AxyPrep Plasmid Miniprep Kit (Axygen Biosciences), and eluted in 50 μl of the manufacturer's elution buffer, stored at –80°C. Inserted DNA was sequenced by Wuhan TsingKe Biological Technology Co. Ltd to confirm whether consistent with the target DNA. The concentration (ng/μl) of the standards was determined by spectrophotometric measurement (NanoDrop 2000c, Thermo Fisher Scientific Inc.). The copy number of the target DNA fragment was calculated using the following equation: copy number of target DNA fragment = [DNA mass (g)/recombinant plasmid molar mass] × 6.02 × 10^23^ (Wang et al., 2013). A tenfold gradient dilution of standards ranging from 1.62 × 10^9^ to 1.62 × 10^0^ copies/μl was also used to evaluate the sensitivity of the assay.

### 2.6 Detection with cPCR

DNA samples (including each feeding interval and a tenfold gradient dilution of standards) were amplified by a MyCycler PCR amplification instrument (Bio‐Rad) using the designed primers described above. The amplification was carried out in a final volume of 20 μl. Each tube contained 10 μl 2 × *EasyTaq* ^®^ PCR SuperMix (Beijing TransGen Biotechnology Co. Ltd), 1 μl sample DNA, 1 μl reverse primer (10 μM), 1 μl forward primer (10 μM), and 7 μl ultra‐pure water. The thermal cycle consisted of an initial step of 5 min at 94°C, followed by 35 cycles of 30 s at 94°C, 30 s at 57.6°C, 30 s at 72°C, and a final extension step of 5 min at 72°C. Each run contained a negative control (adult *P. pseudoannulata*, starved at least a week) and a non‐template control (without any nucleic acid). Each sample was assayed in triplicate and used to evaluate reproducibility of the assay. PCR products were visualized using agarose gel electrophoresis with 1 × TAE buffer [50 × TAE: glacial acetic acid 57.1 ml, EDTA 100 ml (0.5 M), Tris 242 g, pH 8.0, dissolved in distilled water to 1,000 ml] and 1.5% agarose gel. The band on the agarose gel was photographed using a ChemiDoc™ XRS Imaging Systems (Bio‐Rad) after ethidium bromide staining.

### 2.7 Detection with TaqMan qPCR

DNA samples (including each feeding interval and a tenfold gradient dilution of standards) were amplified by a CFX Connect™ Real‐Time PCR Detection System. The same primers described above were used in the qPCR. qPCR conditions and thermal cycle were as described 2.4. Each run contained a negative control and a non‐template control as described above. Each sample was also assayed in triplicate and used to evaluate reproducibility of the assay. Data acquisition and analysis were as described above.

## 3 RESULTS

### 3.1 The annealing temperature and specificity of primers

**FIGURE 1:**
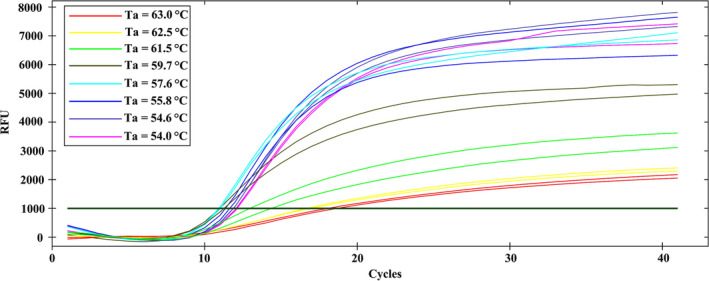
Determining the optimum annealing temperature (Ta) of the primers using a temperature gradient PCR. The same color of the fluorescence curve depicts the same DNA samples

The temperature gradient PCR was performed using a CFX Connect™ Real‐Time PCR Detection System. We set the annealing temperature to 63.0°C, 62.5°C, 61.5°C, 59.7°C, 57.6°C, 55.8°C, 54.6°C, and 54.0°C, respectively. The results showed that the *Ct* value (qPCR cycle number where the fluorescence curve crosses threshold line) was minimized when the annealing temperature was 57.6°C, indicating amplification efficiency of the primers was optimal (Figure 1).

**FIGURE 2:**
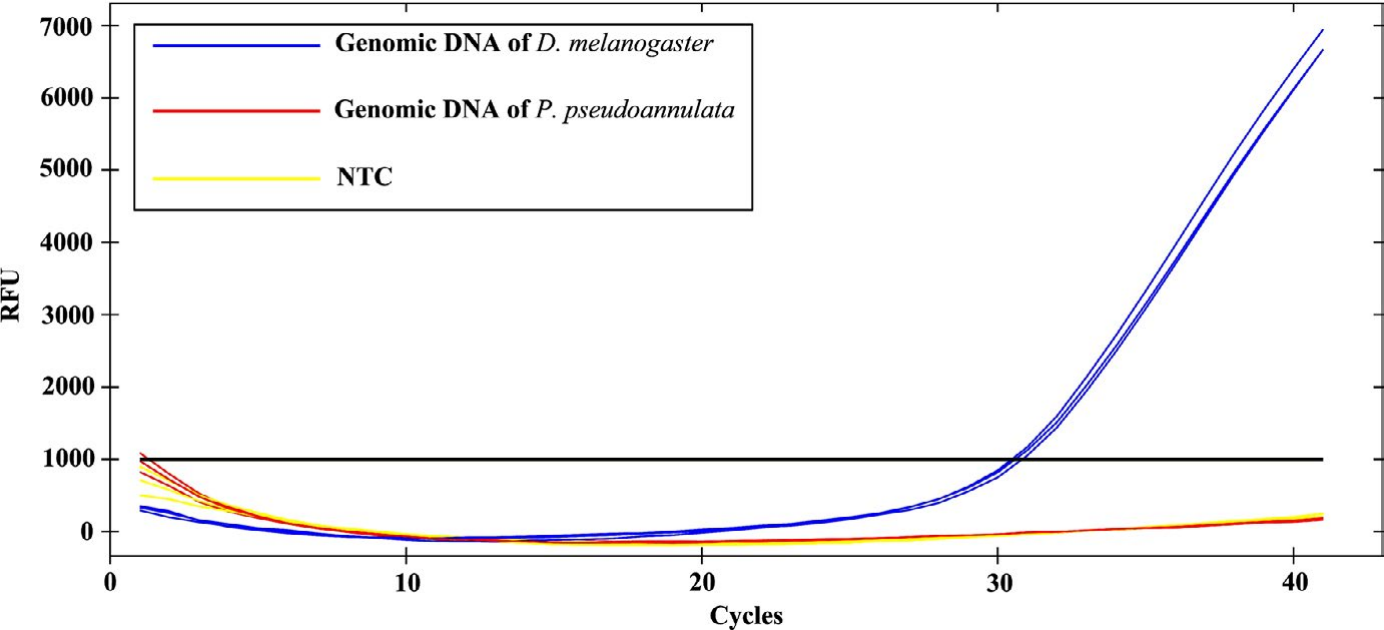
Testing the specificity of the primers using genomic DNA from *Drosophila melanogaster* and *Pardosa pseudoannulata* (starved at least a week). NTC: Non‐template control

The primers COI‐F and COI‐R successfully amplified a 99 bp target DNA fragment of *D. melanogaster* by qPCR, but did not amplify the non‐target genomic DNA of *P. pseudoannulata* (Figure 2).

### 3.2 Sequencing the DNA inserted into the plasmid

**FIGURE 3:**
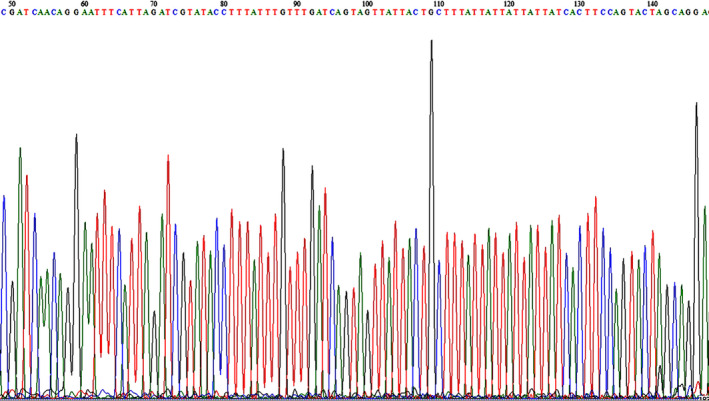
Sequence of DNA inserted into the plasmid

To confirm whether the inserted DNA was consistent with the target DNA, the recombinant plasmid DNA was sequenced by sequencing company. The results showed that the inserted DNA (Figure 3) was 100% of matching with the target DNA from GenBank (GenBank accession no. EF153615.1).

### 3.3 Comparison of sensitivity between cPCR and qPCR

**FIGURE 4:**
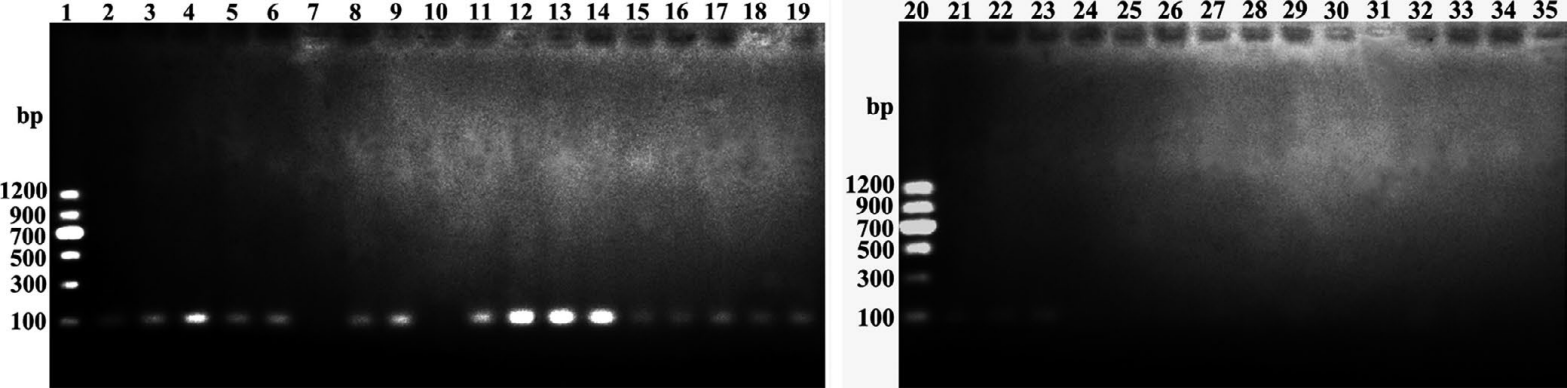
Agarose gel electrophoresis of cPCR‐amplified DNA of tenfold gradient dilution of standards ranging from 1.62 × 10^9^ to 1.62 × 10^0^ copies/μl. Cropped gels are merged and displayed (the full‐length of each uncropped gel is 11.5 cm). Lane 1 and Lane 20: DNA marker (Marker II, Tiangen Biotech (Beijing) Co., Ltd.); Lanes 2–4:1.62 × 10^9^ copies/µl; Lanes 5–7:1.62 × 10^8^ copies/µl; Lanes 8–10:1.62 × 10^7^ copies/µl; Lanes 11–13:1.62 × 10^6^ copies/µl; Lanes 14–16:1.62 × 10^5^ copies/µl; Lanes 17–19:1.62 × 10^4^ copies/µl; Lanes 21–23:1.62 × 10^3^ copies/µl; Lanes 24–26:1.62 × 10^2^ copies/µl; Lanes 27–29:1.62 × 10^1^ copies/µl; Lanes 30–32:1.62 × 10^0^ copies/µl; Lanes 33–35: NTC

**FIGURE 5:**
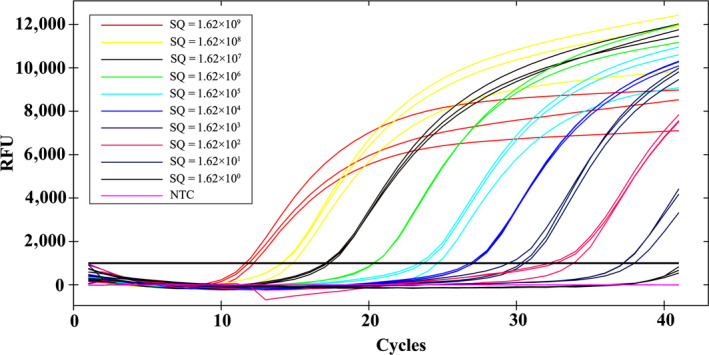
The fluorescence curve of TaqMan qPCR‐amplified DNA of a tenfold gradient dilution of standards ranging from 1.62 × 10^9^ to 1.62 × 10^0^ copies/μl. RFU: relative fluorescence units; SQ: starting quantity

The sensitivity of the assays was evaluated using DNA samples from adult female *P. pseudoannulata* at various time periods after the consumption of three adult *D. melanogaster*, and a tenfold gradient dilution of standards ranging from 1.62 × 10^9^ to 1.62 × 10^0^ copies/μl. The results showed that the cPCR assays could detect as few as 1.62 × 10^3^ copies of the target DNA fragment. No band was observed on the agarose gel if the concentration of standards ranged from 1.62 × 10^2^ to 1.62 × 10^0^ copies/μl (Figure 4). However, the TaqMan qPCR assays could detect as few as 1.62 × 10^1^ copies of the target DNA fragment. The fluorescence curve was less obvious only when the concentration of standards was 1.62 × 10^0^ copies/µl (Figure 5). The cPCR assays showed that the positive rate of target DNA fragment was 100% after 0 hr of digestion, decreasing to 80% after 48 hr of digestion. The positive rate decreased to 0 after 72 and 96 hr of digestion (Table 1). However, the TaqMan qPCR assays showed that all spiders were positive after consuming prey at various time intervals (0, 24, 48, 72 and 96 hr) (Table 1). These results showed that the sensitivity of qPCR was obviously higher than cPCR in molecular gut content analysis.

**TABLE 1:** The sensitivity of cPCR and TaqMan qPCR

Hours post‐feeding (hr)	Individual number of spiders	Positive rate of target DNA fragment*	
		cPCR test	qPCR test
0	5	100% (5)^#^	100% (5)
24	5	100% (5)	100% (5)
48	5	80% (4)	100% (5)
72	5	0 (0)	100% (5)
96	5	0 (0)	100% (5)

Note

The assay was evaluated using DNA of adult female *Pardosa pseudoannulata* individuals at various time periods after the consumption of three adult *Drosophila melanogaster*.

* The DNA sample is considered positive if one of the three technical replicates is positive. The DNA sample is considered negative if all three technical replicates are negative.

^#^ Data in brackets is the number of tested positive for each post‐feeding treatment.

### 3.4 Comparison of reproducibility between cPCR and qPCR

**FIGURE 6:**
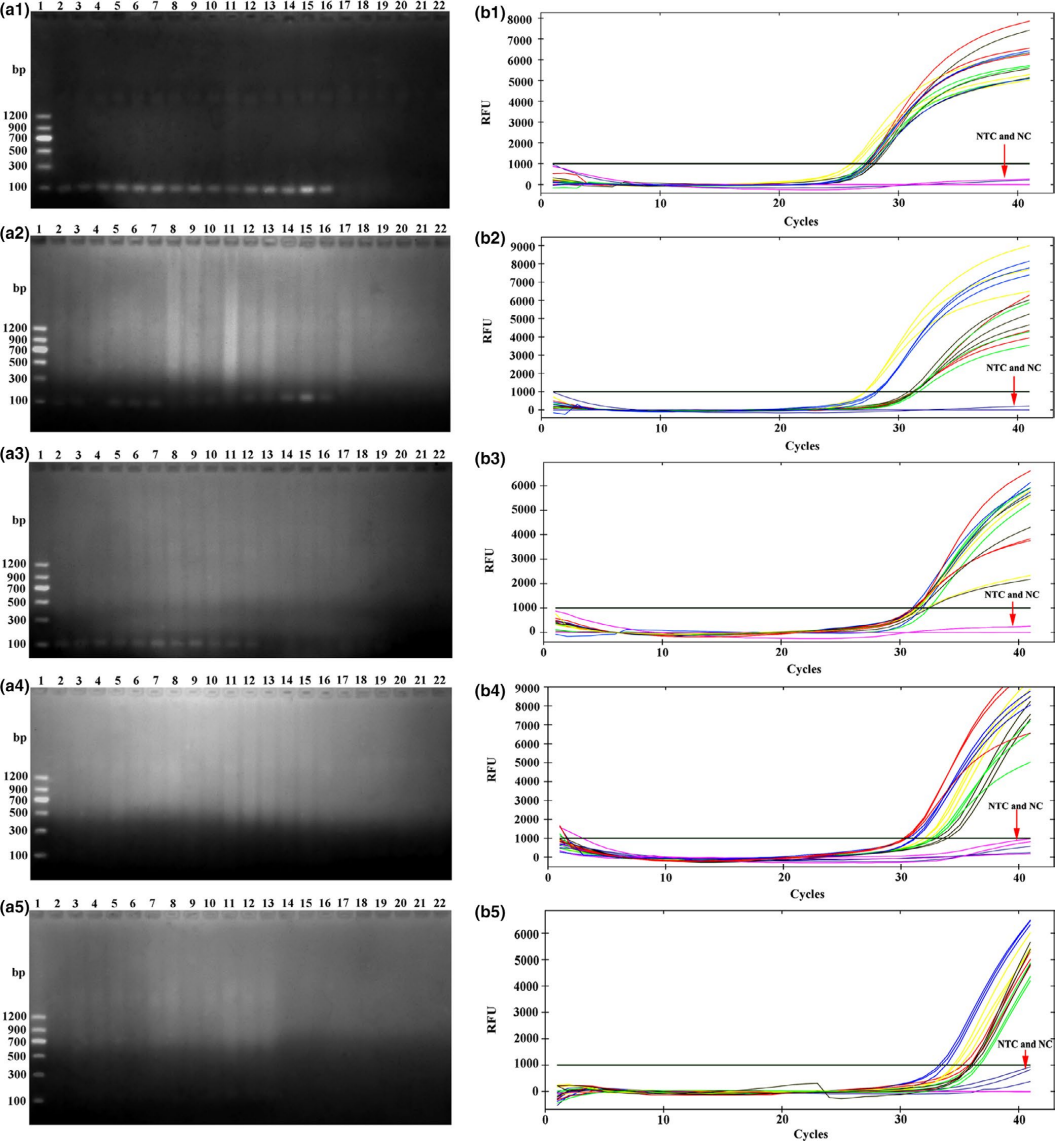
Evaluating the sensitivity and reproducibility of the assays using DNA samples from adult female *Pardosa pseudoannulata* at various time periods after the consumption of three adult *Drosophila melanogaster*. Each sample was assayed in triplicate. (a1)–(a5): Agarose gel electrophoresis of cPCR‐amplified DNA samples. Cropped gels are merged and displayed (the full‐length of each uncropped gel is 11.5 cm). Lane 1: DNA marker (Marker Ⅱ, Tiangen Biotech (Beijing) Co., Ltd.); Lane 2–Lane 16: DNA samples from feeding trials (Lane 2–Lane 4: Sample DNA 1; Lane 5–Lane 7: Sample DNA 2; Lane 8–Lane 10: Sample DNA 3; Lane 11–Lane 13: Sample DNA 4; Lane 14–Lane 16: Sample DNA 5); Lane 17–Lane 19: NTC; Lane 20–Lane 22: Negative control (NC). (b1)–(b5): The fluorescence curve of TaqMan qPCR‐amplified DNA samples. The same color of fluorescence curve depicts the same DNA samples. (a1) and (b1): 0 hr post‐feeding; (a2) and (b2): 24 hr post‐feeding; (a3) and (b3): 48 hr post‐feeding; (a4) and (b4): 72 hr post‐feeding; (a5) and (b5): 96 hr post‐feeding

The reproducibility of the assays was evaluated using technical replicates of each DNA sample. Among the 21 positive DNA samples (including DNA samples from adult female *P. pseudoannulata* at various time periods after the consumption of three adult *D. melanogaster*, and a tenfold gradient dilution of standards ranging from 1.62 × 10^9^ to 1.62 × 10^0^ copies/μl), only 14 DNA samples showed that all three technical replicates were positive with cPCR. Moreover, some bands on the agarose gel were absent or gray, while some were white and bright for the same DNA samples after amplification by cPCR (Figures 4 and 6). However, among the 34 positive DNA samples, a total of 33 DNA samples showed that all three technical replicates were positive with the TaqMan qPCR (Figures 5 and 6). Moreover, the coefficients of variation of the *Ct* value for the three technical replicates of each DNA sample were less than 5% (0.04%–2.86%) (Tables 2 and 3). These results showed that the reproducibility of qPCR was obviously higher than that of cPCR in molecular gut content analysis.

**TABLE 2:** The reproducibility of TaqMan qPCR

Copy number of target DNA fragment	*Ct*	*CV* (%)
1.62 × 10^9^	11.90 ± 0.29	2.44
1.62 × 10^8^	14.34 ± 0.41	2.86
1.62 × 10^7^	17.19 ± 0.14	0.81
1.62 × 10^6^	20.32 ± 0.03	0.15
1.62 × 10^5^	23.89 ± 0.67	2.80
1.62 × 10^4^	27.00 ± 0.12	0.44
1.62 × 10^3^	30.11 ± 0.57	1.89
1.62 × 10^2^	32.89 ± 0.85	2.58
1.62 × 10^1^	37.49 ± 0.44	1.17
1.62 × 10^0^	—	—

Note

The assay was evaluated using a tenfold gradient dilution of standards ranging from 1.62 × 10^9^ to 1.62 × 10^0^ copies/μl. *Ct* values are presented as the mean ± *SD* (*N* = 3). *CV*: coefficients of variation.

**TABLE 3:** The reproducibility of TaqMan qPCR

Hours post‐feeding (hr)	Sample DNA 1		Sample DNA 2		Sample DNA 3		Sample DNA 4		Sample DNA 5	
	*Ct*	*CV* (%)	*Ct*	*CV* (%)	*Ct*	*CV* (%)	*Ct*	*CV* (%)	*Ct*	*CV* (%)
0	27.36 ± 0.17	0.62	26.15 ± 0.19	0.73	27.80 ± 0.34	1.22	27.36 ± 0.33	1.21	27.46 ± 0.26	0.95
24	31.27 ± 0.09	0.29	27.19 ± 0.01	0.04	31.19 ± 0.30	0.96	31.42 ± 0.24	0.76	28.19 ± 0.12	0.43
48	31.25 ± 0.14	0.45	31.68 ± 0.44	1.39	31.78 ± 0.62	1.95	31.81 ± 0.55	1.73	31.39 ± 0.44	1.40
72	30.32 ± 0.11	0.36	32.14 ± 0.22	0.68	33.53 ± 0.47	1.40	32.75 ± 0.16	0.49	30.94 ± 0.37	1.20
96	35.64 ± 0.34	0.95	34.73 ± 0.33	0.95	36.07 ± 0.08	0.22	36.37 ± 0.38	1.04	33.53 ± 0.30	0.89

Note

The assay was evaluated using DNA of adult female *Pardosa pseudoannulata* individuals at various times after the consumption of three adult *Drosophila melanogaster*. *Ct* values are presented as the mean ± *SD* (*N* = 3).

## 4 DISCUSSION

Studies of predation is one of the highlights of ecological research (Bael et al., 2008; Ge, 2008; Holtgrieve, Schindler, & Jewett, 2009). In this process, it is particularly important to choose applicable methods to identify predator–prey interactions in agricultural landscapes, or agricultural fields. Molecular gut content analysis is a common method, which is more practical than previous methods (direct observation (Heimpel, Rosenheim, & Mangel, 1997), camera trapping (Foster et al., 2013), gut dissection (Triltsch, 1997), isotope labeling (Crossley, 1966; Wang, Jiang, & Zhang, 2015), chromatography (Sloggett, Obrycki, & Haynes, 2009), electrophoresis analysis (Camara, Borgemeister, Markham, & Poehling, 2003), and the use of monoclonal antibodies (Griffiths et al., 2008)). This is particularly the case for studying the predation of nocturnal predators and some relatively small arthropod predators, which are difficult for direct observation predation in the field (King et al., 2008). PCR is sensitive, specific, and reproducible and can be used to analyze the DNA of the prey remains in the gut of predator. To date, both cPCR and qPCR have been successfully employed to identify predator–prey relationships in the field (Li et al., 2017; Yang, Liu, Yuan, Zhang, Li, et al., 2017; Yang, Liu, Yuan, Zhang, Peng, et al., 2017). We used feed trial experiments to further demonstrate which method was more ideal for detecting predation in the field.

We used the COI gene of *D. melanogaster* to screen *D. melanogaster‐*specific DNA fragment, and a tenfold gradient dilution of standards was constructed using the specific DNA fragment. Additionally, we obtained DNA samples from *P. pseudoannulata* individuals that fed on *D. melanogaster* at post‐feeding intervals of 0, 24, 48, 72 and 96 hr. Finally, we compared the sensitivity and reproducibility between cPCR and qPCR assays for detecting DNA samples from feeding trials and standards. The results showed that the cPCR assays could detect as few as 1.62 × 10^3^ copies of the target DNA fragment. However, the TaqMan qPCR assays could detect as few as 1.62 × 10^1^ copies of the target DNA fragment. The cPCR assays could detect as few as 48 hr post‐feeding of the target DNA fragment. However, the TaqMan qPCR assays showed that all spiders were positive after consuming prey at various time intervals (0, 24, 48, 72 and 96 hr). These results showed that the sensitivity of qPCR was obviously higher than that of cPCR in molecular gut content analysis. This is consistent with the findings of Gomez‐Polo et al. (2015), in which qPCR is more sensitive than cPCR in detecting *Nasonovia ribisnigri* DNA remains in the gut of *Episyrphus balteatus* using *N. ribisnigri*‐specific primers (154 bp). In terms of reproducibility, it has still not been reported for comparing the reproducibility of cPCR and qPCR in molecular gut content analysis. Although post‐visualization method of cPCR (based on agarose gel) could be minimizing or hampered the detection compared to other visualization methods of cPCR, such as post‐PCR visualization using a capillary electrophoresis system (Sint, Raso, Kaufmann, & Traugott, 2011). However, our results showed that a smaller proportion of the technical replicates were positive using cPCR and some bands on the agarose gel were absent or gray, while some were white and bright for the same DNA samples after amplification by cPCR. By contrast, a larger proportion of the technical replicates were positive using qPCR and the coefficients of variation of the *Ct* value for the three technical replicates of each DNA sample were less than 5%. These results showed that the reproducibility of qPCR was obviously higher than that of cPCR in molecular gut content analysis.

The prey DNA was broken into smaller fragments during digestion in the predator's guts (King et al., 2008). Thus, the prey DNA that remains in the gut or feces of predators was usually low‐quality DNA samples. Additionally, the detectability of prey DNA remains depended on predator species (fundamental dissimilarities in prey digestion capacities), ambient temperature (high temperatures significantly decreased detection rates), time since feeding (detection rates significantly decreased with increasing digestion time), and meal size (the more prey eaten by a given predator species the greater the probability of obtaining a positive reaction) (Eitzinger, Unger, Traugott, & Scheu, 2014; Hagler & Naranjo, 1997; Leal, Nejstgaard, Calado, Thompson, & Frischer, 2014; Von Berg, Traugott, Symondson, & Scheu, 2008; Weber & Lundgren, 2009). For these problems, higher sensitivity detection technology may be necessary. The present study provides an example of the use of cPCR and qPCR to detect the target DNA fragment of prey remains in predator's gut. It is also shown that qPCR is more sensitive and highly reproducible in detecting such degraded DNA. In addition, the products of qPCR could generate results by a fluorescence curve without further treatment, which greatly saved time and reduced the possibility of carryover contamination (Balamurugan et al., 2009). The generated *Ct* value could be used for quantitative analysis of template DNA, which was significant in quantitatively evaluating the predation of insect pests by predatory natural enemies (Yang, Liu, Yuan, Zhang, Li, et al., 2017; Yang, Liu, Yuan, Zhang, Peng, et al., 2017).

In recent years, the diet composition of predators has been analyzed using next‐generation sequencing (Biffi et al., 2017; Crisol‐Martinez, Moreno‐Moyano, Wormington, Brown, & Stanley, 2016; Krehenwinkel, Kennedy, Pekar, & Gillespie, 2017; Piñol, San Andrés, Clare, Mir, & Symondson, 2014; Pompanon et al., 2012; Zhong, Tan, Wang, & Yan, 2019). Unlike cPCR and qPCR, the DNA fragments of various prey species can be amplified in a single reaction based on general primers of potential prey. This method is suitable for analyzing the diet composition of generalist predators (e.g., spiders) (Krehenwinkel et al., 2017; Lafage et al., 2019). However, this technology can only be as precise as the sequence databases of species barcode available. At present, most of the sequencing results are identified only at the level of families or genera (Piñol et al., 2014; Zhong et al., 2019). Therefore, qPCR is the preferred method only if studying the predation of target prey by predators. The results are accurate based on the prey‐specific primers, and the amount of template DNA can be quantified using qPCR.

## CONFLICT OF INTEREST

The authors declare no competing or financial interests.

## AUTHOR CONTRIBUTIONS

**Ting‐bang Yang:** Conceptualization (equal); formal analysis (equal); funding acquisition (equal); investigation (equal); methodology (equal); writing–original draft (equal). **Jie Liu:** Formal analysis (equal); investigation (equal); methodology (equal); writing‐original draft (equal). **Jian Chen:** Conceptualization (equal); funding acquisition (equal); supervision (equal); writing–original draft (equal).

## Data Availability

Raw data of qPCR test are available online on Dryad repository (https://doi.org/10.5061/dryad.1rn8pk0qz).
